# Fishmeal, plant protein, and fish oil substitution with single-cell ingredients in organic feeds for European sea bass (*Dicentrarchus labrax*)

**DOI:** 10.3389/fphys.2023.1199497

**Published:** 2023-05-15

**Authors:** A. Vasilaki, E. Mente, E. Fountoulaki, M. Henry, C. Nikoloudaki, P. Berillis, K. Kousoulaki, I. Nengas

**Affiliations:** ^1^ Hellenic Centre for Marine Research (HCMR), Institute of Marine Biology, Biotechnology and Aquaculture (IMBBC), Attica, Greece; ^2^ School of Veterinary Medicine, Aristotle University of Thessaloniki, Thessaloniki, Greece; ^3^ Department of Ichthyology and Aquatic Environment, School of Agricultural Sciences, University of Thessaly, Volos, Greece; ^4^ Department of Nutrition and Feed Technology, Fyllingsdalen, Norway

**Keywords:** organic feed, single cell ingredients, bacterial protein, yeast protein, microalgae, fishmeal replacement, European sea bass (*Dicentrarchus labrax*)

## Abstract

Single-cell ingredients (SCI) are considered promising nutrient sources which are produced using environmentally friendly biotechnological processes. The aim of the current study was to evaluate the replacement of fishmeal, plant protein sources, and fish oil with SCI in organic feeds for European sea bass (*Dicentrarchus labrax*). Bacterial protein, yeast protein, and microalgae were used to replace fishmeal trimmings, soya bean meal, and fish oil from trimmings. Triplicate groups (30 fish per replicate) of European sea bass (14.4 ± 2.4 g) were fed the experimental diets for 71 days. The results showed that the incorporation of SCI at all levels of inclusion significantly enhanced nutrient digestibility. Additionally, growth performance parameters were not affected by SCI inclusion, exhibiting similar or improved values. Moreover, a tendency for improved anterior and posterior gut structure was observed and a significant *increase of lysozyme activity at the two highest inclusion levels of SCI was determined. Overall, the results showed that the inclusion of SCI at 15%* (bacterial: yeast: algae—9.4: 4.7: 1) is possible without compromising any of the parameters evaluated. According to these findings, a higher substitution of fishmeal trimmings, plant protein sources, and fish oil from trimmings with SCI in organic diets for European sea bass (*D. labrax*) can be further evaluated in future studies.

## 1 Introduction

The human population is increasing globally along with the demand for sustainable high-quality protein sources in order to cover its nutritional requirements. Aquaculture is the food industry that will provide future generations with healthy and environmentally friendly protein. The production of aquaculture species in Europe increased by 17% in the last decade according to EC (2022b). In the Mediterranean region, the main farmed species are gilthead seabream and European seabass with a production volume of 93.000 and 80.000 tonnes/year, respectively, for 2022 [EC (2022b)]. Following the global trend for organic food consumption, the concerns about the declining wild aquatic species populations, seafood safety, traceability, and environmental challenges, organic aquaculture have started gaining the attention of consumers during the last decade. According to EC (2022a), organic aquaculture production in the EU has increased significantly in recent years. However, this growth is mainly attributed to mussel production. The same report also states that organic finfish production is stagnant due to low market demand and production challenges. In 2020, the total organic aquaculture production in the EU was estimated at 86,000 tones, which accounts for only 8% of the total EU aquaculture production EC (2022a). The only finfish species with an increasing production trend are gilthead seabream and European seabass, mostly produced in Greece (EC, 2022a). Organic aquaculture is also driven by the increasing interest in the sustainable use of resources and reflects an alternative farming approach (Lembo and Mente, 2019). This farming strategy supports the natural feeding procedure of farmed fish, ensuring the fulfillment of their behavioral and physiological needs. Additionally, organic feeds must meet the nutritional requirements of farmed organisms, securing optimum production performance, health, welfare, and high quality of the final product while leaving a low environmental footprint (Busacca and Lembo, 2019). Fishmeal and fish oil contain many indispensable nutrients and components, such as protein and amino and fatty acids, playing a significant role in the feed formulations of conventional and organic aquafarming (Kaushik and Seiliez, 2010; Krogdahl et al., 2010; Lund et al., 2013). However, organic and conventional farming strategies face the same challenges since fishmeal is not sustainable in the long term (Turchini et al., 2019). The use of fishmeal and fish oil is against the principles of organic aquaculture, due to the overexploitation of wild stocks. In contrast, the use of fishmeal trimmings and fish oil trimmings is permitted when wild fish are harvested for human consumption. Fishmeal trimmings consist of by-products from the industrial-scale processing of fish for human consumption (Stevens et al., 2018). However, concerns are growing due to the high phosphorus content in fishmeal trimming and the environmental regulations (Lembo and Mente, 2019). Fishmeal and fish oil production produced from processing by-products reached 27% and 48% of global production, respectively (www.iffo.com). Fishmeal from trimmings has different nutritional value compared to conventional fishmeal with lower protein levels but higher minerals concentration (Dam et al., 2019; Kandyliari et al., 2020). Even though the nutritional quality of fishmeal and fish oil trimmings is considered promising, the actual demand for fishmeal from the aquaculture industry is rather high and this inevitably leads to the search and incorporation of novel raw materials with high nutritious value in aqua feed formulations.

Considerable research has been performed on plant protein sources showing successful outcomes in diets for herbivorous species as they possess the requisite enzymes to utilize plant-based proteins (Hlophe et al., 2014). However, plant protein sources for carnivorous species increase the possibility for nutritional deficiencies and contain several antinutritional factors such as trypsin inhibitors, phytic acid, and fibers that decrease the nutrient absorption and digestibility of feeds (Collins et al., 2013; Dawood and Koshio, 2020). Innovative processing techniques aiming to improve the nutritional quality of plant ingredients have been used during the last decade. Fermentation technology is considered one of these and is capable of reducing antinutritional factors of plant ingredients and increasing their protein content.

Such technologies have been also used for treating and producing novel feed ingredients such as single-cell proteins. Algae, bacteria, fungi, and yeast can produce single-cell proteins with the use of an appropriate substrate. The inclusion of single-cell ingredients in aqua feed formulations is rising (Albrektsen et al., 2022) and is expected to play a significant role in future aquaculture sustainability. The use of efficient and suitable ingredients for aquaculture feeds is a critical issue for European fish farming because of its direct influence on production and profit.

The production of microbial ingredients with biotechnological processes by waste or methanol oxidation is an approach attempted many decades ago (Westlake, 1986). The rapid growth of bacteria on organic substrates is a characteristic that makes them ideal candidates for the production of novel protein raw materials. The protein content of such products can reach up to 80% on dry matter and can be a source of minerals, nucleic acids, and vitamins (Biswas et al., 2020). Methanotroph bacteria, such as *Methylococcus capsulatus*, have the ability to use methane as an energy source with gas-based fermentation technology. The amino acid profile and nutrient content of such protein sources are comparable to the fishmeal profile and other protein raw materials used in aqua feeds (Sharif et al., 2021). Microbial proteins have been studied for several years with controversial results. In rainbow trout, it was reported that up to 80% of fishmeal can be replaced by microbial proteins (Kaushik and Luquet, 1980) but in a study by Kiessling and Askbrandt (1993) poor digestibility of protein was observed, leading to lower fish growth. Since then, several bacteria strains were used for the production of microbial proteins with an emphasis on methanotrophic bacteria which have the potential to convert 70%–90% of the methane (otherwise released in the atmosphere) to over 60 million tonnes of protein (Kalyuzhnaya et al., 2013). The use of microbial protein, in recent studies, showed high growth rates and digestibility in several other species (Biswas et al., 2020; Zamani et al., 2020; Biswas et al., 2021; Pilmer et al., 2022).

Single-cell ingredients (SCI) derived from yeast, such as *Saccharomyces cerevisiae*, have a high crude protein content and are considered a rich source of dietary nucleotides, which can enhance immune function and growth. This form of yeast extract has been evaluated as a fishmeal replacement in several species, with positives effects (McLean and Craig, 2004; Lunger et al., 2006; Lunger et al., 2007; Peterson et al., 2012; Panagiotidou et al., 2016).

Microalgae can reinforce sustainability in aquaculture as a potential alternative ingredient to fish oil and protein. Marine microalgae, *Schizochytrium* sp., is considered a promising source of n-3 long-chain polyunsaturated fatty acids (LC-PUFAs) due to its high content of docosahexaenoic acid (DHA) that can reach up to 22% of dry matter (Bélanger et al., 2021). This species of algae meal is produced heterotrophically using fermentation technology and is free from pollutants. Studies have confirmed the successful replacement of fish oil by this source of n-3 LC-PUFAs in fillet tissue of channel catfish, Atlantic salmon, and Nile Tilapia (Li et al., 2009; Kousoulaki et al., 2015; Kousoulaki et al., 2016; Sarker et al., 2016).

All the above-mentioned non-conventional protein and lipid sources can constitute possible novel ingredients of raw materials to meet the needs of the rapid development of the aquaculture industry contributing to the circular economy in a sustainable way. The aim of this study was the evaluation of SCI (microbial protein, yeast protein, and algae meal) as replacements for fishmeal trimmings, plant protein ingredients, and fish oil from trimmings in organic feeds for European sea bass (*Dicentrarchus labrax*).

## 2 Materials and methods

### 2.1 Fish husbandry

European sea bass juveniles (*D. labrax*) of initial average body weight of 14.4 ± 2.4 g were supplied from a commercial fish farm in Greece and transported to the facilities of the laboratory of Applied Fish Nutrition, Institute of Marine Biology, Biotechnology and Aquaculture, of Hellenic Centre for Marine Research in Athens (license number EL 25 BIO 37, 498/05-02-2018l). The fish were acclimatized for a period of 20 days and distributed in 12 cylindoconical tanks with a 250 L capacity. In total, 30 fish per tank were placed in triplicate groups for each diet. The tanks were equipped with fecal traps, using a modification of the Guelph method (Cho et al., 1982). Feces were collected into 50 mL falcon tubes and the samples were centrifuged and stored in the freezer (−20°C), and then freeze-dried and analyzed for nutrient apparent digestibility coefficient (ADC). Ten fish were randomly collected from the initial fish population and euthanized using an overdose of anesthesia (clove oil with ethanol 1:10), minced, and freeze-dried, for whole-body composition, fatty acid, and amino acid profiles. During the experiment all the fish were subjected to a photoperiod of 12L: 12D to simulate dawn and dusk, seawater salinity was 38 ppt and the temperature was kept at 22°C ± 1°C. The temperature and dissolved oxygen (DO) were recorded daily. Total ammonia (NH_3_/ ${NH}_{4}^{+}$ ), nitrate ( ${NO}_{3}^{-}$ ), and nitrite ( ${NO}_{2}^{-}$ ) were measured daily using API tests (aquarium pharmaceuticals) and never exceeded 0.25, 0.25, and 1.0 ppm, respectively. The fish were fed the experimental diets by hand three times per day to apparent satiation until the end of the trial. The duration of the experimental trial was 71 days. Feces were collected daily from 15 days before the trial expiration for the determination of nutrient ADC of the diet. Mortalities and feed consumption were recorded daily. All the groups of fish were fasted 1 day before samplings. Fish were anesthetized using diluted 1:10 clove oil with ethanol and weighed individually at the beginning, the middle, and at the end of the experimental period.

### 2.2 Experimental diets

Four isonitrogenous and isoenergetic experimental diets (Table 1) were designed and produced at the facilities of the Applied Nutrition lab of the Institute of Marine Biology, Biotechnology and Aquaculture in Athens, using a lab scale twin-screw extruder (model EV025A107FAA, CLEXTRAL) and oil was added by vacuum coating (DINISSEN). A control diet was formulated with organic raw materials (fishmeal trimmings, soybean meal, and fish oil from trimmings) to imitate the composition of a commercial organic diet. Three experimental diets were formulated with the inclusion of SCI consisting of bacterial protein (FeedKind^®^ by Calysta Inc.), yeast protein (NuPro^®^ by Alltech), and algae meal (AlgaPrime™ DHA by Corbion) in different levels of inclusion. The proximate composition of SCI is presented in Table 2. Specifically, diet SCI 12 had a 12% inclusion of SCI (bacterial: yeast: algae—7.5: 3.8: 1), diet SCI 15 had a 15% inclusion of SCI (9.4: 4.7: 1) and diet SCI 18 had an 18% inclusion of SCI (11.3: 4.6: 1). To all diets with SCI incorporation, organic soya bean meal was eliminated and soy protein concentrate was noticeably decreased. Additionally, fish oil from trimmings was decreased and rapeseed oil increased. Krill meal was incorporated in diets SCI 12, SCI 15, and SCI 18 to meet the requirements of essential fatty acids of sea bass as fishmeal trimmings and fish oil were reduced in those diets and additionally prevent possible palatability issues. Vitamins and minerals were added to all diets. Yttrium oxide (Y_2_O_3_) was incorporated in all experimental diets as an inert marker to enable ADC determination (Table 1). The amino acid and fatty acid profiles of the experimental diets are given in Tables 3 and 4, respectively.

**TABLE 1:** Diet formulation and proximate composition of the experimental diets (%).

	Control	SCI 12	SCI 15	SCI 18
Fishmeal trimmings 65	20	18.8	15.9	13.1
Krill meal	—	5	5	5
Bacterial protein	—	7.5	9.4	11.3
Yeast protein	—	3.8	4.7	5.6
Algae meal	—	1	1	1
Wheat meal	10.6	17.4	16.8	16.1
Wheat gluten	6.0	11.0	10.0	11.0
Corn gluten	15.0	12.5	11.0	11.0
Organic soybean meal	8.0	—	—	—
Soy protein concentrate	24.0	8.0	11.0	10.0
Trimmings fish oil	11	5	5	5
Rapeseed oil	3	7.0	7.0	7.2
Monocalcium Phosphate	1.5	1.8	1.8	2.1
Mineral premix^a^	0.2	0.2	0.2	0.2
Vitamin premix^b^	0.2	0.2	0.2	0.2
Lysine	0.3	0.8	0.9	1.1
Methionine	0.2	0.1	0.1	0.1
Yttrium oxide	0.1	0.1	0.1	0.1
Dry matter	95.1	94.8	94.8	95.3
Protein	46.9	46.7	47.1	46.9
Fat	18.9	19.0	17.9	17.7
Ash	7.3	7.1	6.9	6.8
Starch	10.0	14.0	14.4	13.4
Energy kJ/g	22.4	22.3	22.2	22.2

^b^ Vitamin premix consisted of: biotin; folic acid; niacin; pantothenic acid; pyridoxine; riboflavin; thiamin; vitamin B12; ascorbic acid; tocopherol acetate; vitamin K.

^a^ Mineral premix consisted of Se; Cu; Mn; Zn in organic form.

**TABLE 2:** Composition of single-cell ingredients (%).

	Protein	Fat	Moisture	Ash
Bacterial protein	69.5	1.0	5.8	7.0
Yeast meal	46.2	0.3	4.7	5.6
Algae meal	12.2	55.7	0.1	5.7

**TABLE 3:** Essential amino acid composition (%) of the experimental diets on a dry weight basis. Data between brackets correspond to the percentage of variation of an AA in diets containing single-cell ingredients with regard to that AA in the control diet.

Amino acids	Control	SCI 12	SCI 15	SCI 18
Arginine	2.5	2.1 (−15.7)	2.2 (−11.9)	2.1 (−15.3)
Histidine	0.9	0.9 (−6.6)	0.8 (−9.7)	0.9 (+3.3)
Isoleucine	1.8	1.8 (−0.3)	1.7 (−5.6)	1.8 (+0.2)
Leucine	4.2	3.9 (−7.1)	3.8 (−9.4)	4.0 (−4.4)
Lysine	2.3	2.5 (+8.0)	2.6 (+12.1)	2.9 (+25.2)
Methionine	1.1	1.1 (−0.3)	1.0 (−8.7)	1.1 (−8.2)
Phenylalanine	2.2	2.1 (−4.7)	2.1 (−4.7)	2.2 (+0.2)
Threonine	1.6	1.7 (+5.5)	1.7 (+5.5)	1.7 (+6.1)
Valine	2.0	2.0 (−0.3)	2.0 (−0.4)	2.1 (+4.9)

**TABLE 4:** Fatty acid composition of the experimental diets (% in diet).

	Control	SCI 12	SCI 15	SCI 18
∑Saturated	3.8	3.7	3.4	3.4
∑Monounsaturated	9.9	10.1	9.4	9.6
∑n9	9.3	9.4	8.7	8.8
∑n6	3.1	3.4	3.1	3.1
∑n3	2.1	2.2	2.2	2.3
EPA	0.7	0.6	0.7	0.7
DHA	0.8	0.9	0.9	0.9
HUFA	1.4	1.5	1.6	1.6
n3/n6	0.7	0.7	0.7	0.7

### 2.3 Growth parameters and biochemical analysis

At the end of the experimental period, all the fish were weighed individually for the evaluation of growth parameters. In total, 20 fish were used for the determination of hepatosomatic and liposomatic indices, and 10 more fish per tank were sampled for whole-body gross composition. Additionally, five fish per tank were used for the determination of whole-body fatty and amino acid profile. Fish for fatty and amino acid estimations were kept at −80°C until analysis.

The following parameters were calculated at the end of the rearing period to compare the key performance indices among the treatments.

Hepatosomatic index: HSI (%) = liver weight (g) ×100/fish weight (g)

Liposomatic index: LSI (%) = visceral fat weight (g) ×100/fish weight (g)

Weight gain: WG = weight final (g)—weight initial (g)

Specific growth rate: SGR (%/day) = ((Ln final weight)− (Ln initial weight)) × 100/trial days.

Feed consumption: FC (%) = ((feed intake g/fishx100)/(initial body weight/fish) × (final body weight/fish)^0.5^))/days of feeding.

Feed conversion ratio: FCR = feed consumed g/growth g

The apparent digestibility coefficient (ADC) of crude protein, crude fat, and starch, was estimated by an indirect method using yttrium oxide as the inert marker by the equation (Cho, 1985):

$$ADC\%=100\;x\;\left(1-\left(Fnutr\;x\;Dy\right)/\left(Dnutr\;x\;Fy\right)\right)$$

where, Fnutr = nutrient concentration in feces, Dnutr = nutrient concentration in diet, Dy = yttrium concentration in diet, and Fy = yttrium concentration in feces.

The proximate composition of feeds, whole body, and freeze-dried feces was determined according to AOAC (2005): ash content by incineration for 12 h at 500°C (Method number 920.153); crude protein (Nx6.25) according to Kjeldahl method (Method number 988.05); and total fat in extruded feeds by acid hydrolysis with HCl followed by ether extraction in a Soxhlet apparatus (SOXTEC SYSTEM HT, 1043 Extraction unit Foss Tecator) according to the ISO method 6492:1999 (Animal Feeding Stuff—Determination of fat content). Total lipids in the feces were determined by the phosphovanillin method (Nengas et al., 1995). Starch was determined by an enzymatic method [Megazyme Total Starch Assay kit (AA/AMG), Megazyme International, Ireland] (McCleary et al., 1994), using thermostable α-amylase and amyloglucosidase. Yttrium was estimated by the ICP-MS method. The fatty acid profiles of the whole body were determined by Gas Chromatography according to Fountoulaki et al. (2009) and amino acid profiles were analyzed by UPLC according to Kotzamanis et al. (2018).

### 2.4 Immunological analysis

At the end of the experimental trial, 6 fish per tank were anesthetized and blood was taken by caudal vein puncture and kept overnight in the fridge to allow coagulation. The next day, samples were centrifuged at 20000 g for 10 min and serum was kept at −80°C until the analysis of the different humoral immune parameters. The serum antibacterial activity of lysozyme against a Gram-positive bacterium, *Micrococcus luteus,* was assessed by the turbidimetric kinetic method as described before (Kokou et al., 2012). The total antibacterial activity of the serum against a luminescent strain of the Gram-negative bacterium *E. coli* was assessed by following the kinetic of luminescence produced by the bacteria in contact with the fish sera (Henry et al., 2009; Henry et al., 2022). The anti-protease activity was expressed as the percentage of inhibition of the trypsin activity on azocasein (Henry and Fountoulaki, 2014). The nitric oxide was measured by the Griess reaction and the ceruloplasmin activity was determined by following the kinetic increase of absorbance at 550 nm due to the oxidation of p-phenylenediamine (Henry et al., 2022).

### 2.5 Histological analysis

Three fish per tank were taken at the end of the experiment for histological analysis. Fish were placed immediately on ice after euthanization and samples of the liver and anterior and posterior gut were taken. Samples were then fixed in 4% formaldehyde for 24 h at 4°C, which were then dehydrated in a graduated series of ethanol, submerged in xylol, and embedded in paraffin wax. Axiostar plus Carl Zeiss Light Microscopy, manufactured by Carl Zeiss Ltd. in Gottingen, Germany, was used to magnify sections of tissue that were 5–7 μm in size and which were deparaffinized, rehydrated, stained with Hematoxylin-Eosin, mounted with Cristal/Mount and examined for alterations beforehand. The histopathological changes of the tissues under study were graded using a semi-quantitative approach (Johnson et al., 2009). The severity grading used the following system: Grade 0 (not remarkable), Grade 1 (minimal), Grade 2 (mild), Grade 3 (moderate), and Grade 4 (severe). The brush border’s width of the anterior gut and the lipid droplets’ diameter were measured from the microscope images according to Berillis et al. (2017).

### 2.6 Statistical analysis

All statistical analyses were carried out using GraphPad Prism 8.4.2. Data are expressed as means ± standard deviations (SD) or standard error of the mean (SEM) of triplicate samples. Normal distribution and homogeneity of variance were checked using Normal Qplot and the Brown-Forsythe test, respectively. One-way ANOVA and Tukey’s *post hoc* test were performed. A Pearson correlation was performed between the fatty acids in the feeds and carcasses of European sea bass. The differences were considered significant when *p* < 0.05.

## 3 Results

### 3.1 Growth parameters and feed utilization

The overall growth performance of European sea bass is given in Table 5. There were no statistically significant differences between the control groups and SCI groups (*p* > 0.05). The groups that were fed the SCI at levels of 12% and 15% showed higher final body weight, however, no significant difference was observed, with the group fed the SCI 15 diet exhibiting a trend for higher final body weight (*p*-value = 0.0814). The population fed a higher level of SCI (SCI 18) exhibited similar final body weights to that fed the control diet. Similarly, experimental fish feed diets with low and moderate inclusion of SCI resulted in higher weight gain compared to control and high inclusion SCI groups. The results for feed conversion ratio (FCR) showed no significant differences among the diets, with values ranging from 1.11 to 1.14. Specific growth rate values were not affected by the incorporation of SCI. A similar trend was observed for the percentage of feed consumption, demonstrating similar values among the dietary treatments. The hepatosomatic index increased with the addition of the SCI but not significantly from the control diet, while no differences were observed in the liposomatic index.

**TABLE 5:** Growth performance of European sea bass.

Parameters	Control	SCI 12	SCI 15	SCI 18
Initial body weight (g)	14.4 ± 0.4	14.5 ± 0.2	14.5 ± 0.2	14.2 ± 0.5
Final body weight (g)	46.3 ± 0.9	49.7 ± 3.0	50.3 ± 1.3	46.9 ± 0.4
Weight gain (g)	32.0 ± 1.2	35.2 ± 2.9	35.8 ± 1.3	32.7 ± 0.9
FCR	1.1 ± 0.0	1.1 ± 0.0	1.1 ± 0.0	1.1 ± 0.0
SGR (%/day)	1.7 ± 0.06	1.7 ± 0.07	1.8 ± 0.04	1.7 ± 0.06
% consumption	2.2 ± 0.0	2.3 ± 0.0	2.3 ± 0.0	2.2 ± 0.1
HSI	1.6 ± 0.1	2.1 ± 0.2	2.0 ± 0.2	2.1 ± 0.4
LSI	4.8 ± 0.3	5.1 ± 0.8	4.8 ± 0.6	4.7 ± 0.3

Data are presented as means of three replicates ±SD, standard deviation.

### 3.2 Nutrient digestibility

Apparent digestibility coefficient values are presented in Table 6. Results showed that the inclusion of SCI significantly affected protein and starch digestibility. Although protein digestibility was high (>93%) for all experimental groups, differences were observed. Protein digestibility in fish fed the control diet was 93.2%, while the corresponding values in fish fed the SCI diets ranged from 93.8% to 94.3%, indicating lower protein digestibility for the control group. Digestibility of fat was similar for all experimental populations with no differences between dietary treatments. However, a trend was observed for higher fat digestibility for the control diet compared to the SCI 18 diet (*p* = 0.077). The inclusion of SCI in European seabass diets was beneficial concerning starch digestibility resulting in higher values. The SCI 15 diet showed significantly higher starch digestibility compared to the SCI 12 and control diets. Similar performance was observed for the group fed the SCI 18 diet, exhibiting a significant difference from the control diet. Furthermore, starch digestibility for the SCI 12 diet was significantly higher than for the control diet.

**TABLE 6:** Nutrient digestibility (ADC) values of feeds (%).

Nutrients	Control	SCI 12	SCI 15	SCI 18
Protein	93.2 ± 0.2^a^	94.3 ± 0.3^b^	94.1 ± 0.1^b^	93.8 ± 0.2^b^
Fat	95.8 ± 0.5	95.6 ± 0.4	95.0 ± 0.2	94.7 ± 0.5
Starch	89.0 ± 0.5^a^	94.1 ± 0.6^b^	95.4 ± 0.4^c^	94.3 ± 0.1 ^bc^

Data are presented as means of three replicates ±SD, standard deviation. Different letters in the same line denote statistically significant differences (*p* < 0.05).

### 3.3 Whole body gross composition, fatty and amino acids profiles

Based on the results presented in Table 7, the inclusion of SCI affected the whole-body composition of the experimental fish. Moisture content was observed to be statistically higher for the groups fed the control, SCI 15, and SCI 18 diets in contrast to the groups fed the SCI 12 diet. Whole-body protein for all dietary treatments showed no differences when compared. However, the whole-body fat content of the groups fed the control diet was significantly lower compared to all the groups fed diets with SCI. For all experimental populations, the ash content was at the same level.

**TABLE 7:** Whole body composition of European sea bass (% wet weight basis).

	Initial population	Control	SCI 12	SCI 15	SCI 18
Moisture	72.9	67.5 ± 0.3^a^	65.3 ± 1.2^b^	65.4 ± 0.8^ab^	66.0 ± 0.7^ab^
Protein	15.3	16.4 ± 0.3	16.8 ± 0.3	16.4 ± 0.4	16.5 ± 0.2
Fat	6.8	11.2 ± 0.1^a^	13.3 ± 1.0^b^	13.2 ± 0.8^b^	12.7 ± 0.4^ab^
Ash	4.2	4.5 ± 0.1	4.3 ± 0.2	4.4 ± 0.1	4.4 ± 0.2

Except initial population all other data are presented as means of three replicates ±SD, standard deviation. Different letters in the same line denote statistically significant differences (*p* < 0.05).

The fatty acid composition of the experimental diets is shown in Table 4. The control diet, with fish oil from trimmings as a source of highly unsaturated fatty acids (HUFA), was determined to have similar levels of EPA and DHA as the diets with SCI where algae meal was incorporated as a source of HUFA to replace fish oil from trimmings. This result was also reflected in the whole-body fatty composition and was validated by a Pearson correlation among feeds and whole bodies (Table 8). Whole-body saturated fatty acids in the groups fed the SCI 12 diet were slightly higher from the rest of the treatments, however, a decreasing trend was observed as SCI inclusion increased. A similar trend was monitored for monounsaturated, total n9, and total n6 fatty acids, where control groups in all cases showed lower levels of these fatty acids. The opposite trend was exhibited for total n3 fatty acids where their levels in the whole body of fish fed the control diet were higher but with no significant differences to those fed the SCI mixture. Significantly higher whole-body EPA levels in the fish fed the control diet were measured in contrast to those fish fed the SCI diets at all inclusion levels. The sum of EPA and DHA showed significant differences between the control diet and the diet with a high inclusion of SCI.

**TABLE 8:** Whole body fatty acid composition of European sea bass (% wet weight basis).

	Control	SCI 12	SCI 15	SCI 18
∑Saturated	3.07 ± 0.31	3.48 ± 0.42	3.35 ± 0.29	3.31 ± 0.44
∑Monounsaturated	6.29 ± 0.56	6.96 ± 0.60	6.77 ± 0.50	6.75 ± 0.44
∑n9	5.85 ± 0.52	6.53 ± 0.54	6.32 ± 0.50	6.29 ± 0.74
∑n6	1.79 ± 0.17	1.94 ± 0.12	1.85 ± 0.09	1.85 ± 0.18
∑n3	1.33 ± 0.17	1.22 ± 0.18	1.18 ± 0.06	1.12 ± 0.12
EPA	0.40 ± 0.02^a^	0.29 ± 0.06^b^	0.28 ± 0.01^b^	0.26 ± 0.04^b^
DHA	0.60 ± 0.04	0.52 ± 0.09	0.50 ± 0.02	0.46 ± 0.05
EPA + DHA	1.00 ± 0.06^a^	0.81 ± 0.15 ^ab^	0.78 ± 0.04 ^ab^	0.72 ± 0.09^b^
^a^Correlation coefficient	**r = 0.99	**r = 0.99	**r = 0.99	**r = 0.99

Data are presented as means of three replicates ±SD, standard deviation. Different letters in the same line denote statistically significant differences (*p* < 0.05).

^a^ Pearson correlation was performed between fatty acids in feeds and carcass.

Regarding the essential amino acid (EAA) composition of the diets (Table 3), the inclusion of SCI led to a deficiency of arginine (12%–16%) compared to the control diet. Histidine also was decreased for the SCI 12 and SCI 15 diets but an excess was observed for the SCI 18 diet (3.3%). Isoleucine was almost equal for all diets except the SCI 15 diet (−5.6%). A deficiency of leucine was shown for all SCI diets (4%–9%). Lysine was added to all the diets and was found to be in excess in diets with SCI (8%–25%). Methionine was equal for all the diets, while threonine was in excess in all SCI treatments. Valine was determined to be at similar levels for all the diets except the SCI 18 diet.

The AA composition in the whole body of European sea bass is presented in Table 9. Significant differences were determined between experimental groups concerning histidine, leucine, and lysine levels. As a general observation, the inclusion of the SCI resulted in a decrease in the EAA for the high inclusion level (SCI 18). The high inclusion of SCI resulted in lower AA content in fish bodies. Specifically, histidine, leucine and lysine were significantly lower compared to the control group. The diets with low or moderate inclusion of SCI showed similar carcass content for all EAAs. Methionine content was similar among all treatments.

**TABLE 9:** Essential amino acid carcass composition of European sea bass (% dry weight basis).

	Control	SCI 12	SCI 15	SCI 18
Arginine	2.63 ± 0.06	2.52 ± 0.07	2.47 ± 0.08	2.47 ± 0.11
Histidine	0.98 ± 0.02^a^	0.93 ± 0.02^ab^	0.92 ± 0.02^ab^	0.91 ± 0.02^b^
Isoleucine	1.78 ± 0.05	1.70 ± 0.06	1.68 ± 0.07	1.61 ± 0.09
Leucine	3.08 ± 0.06^a^	2.92 ± 0.09^ab^	2.91 ± 0.09^ab^	2.82 ± 0.15^b^
Lysine	4.46 ± 0.11^a^	4.23 ± 0.12^ab^	4.21 ± 0.11^ab^	4.07 ± 0.22^b^
Methionine	1.24 ± 0.01	1.18 ± 0.02	1.16 ± 0.04	1.13 ± 0.07
Phenylalanine	1.73 ± 0.03	1.65 ± 0.05	1.64 ± 0.05	1.60 ± 0.08
Threonine	1.85 ± 0.03	1.76 ± 0.04	1.75 ± 0.05	1.72 ± 0.09
Valine	2.08 ± 0.04	1.99 ± 0.04	1.96 ± 0.07	1.92 ± 0.12

Data are presented as means of three replicates ±SD, standard deviation. Different letters in the same line denote statistically significant differences (*p* < 0.05).

### 3.4 Immunology

There were no differences in nitric oxide or ceruloplasmin activity (Table 10), suggesting that no inflammatory response is expected when SCI are added to the fish diet even at the highest dietary level tested. Similarly, complement antibacterial activity was not affected by the experimental diets. Furthermore, the fish feed diets enriched with high levels of bacterial and yeast proteins were significantly immunostimulated with regards to their lysozyme antibacterial activity (from the 15% inclusion level) and anti-protease activity (SCI 18), suggesting a better potential of those fish to fight pathogenic infection. These results could particularly be linked to the inclusion of bacterial and yeast proteins which may immunostimulate the fish.

**TABLE 10:** Immune parameters determined in the fish fed organic ingredients.

	Control	SCI 12	SCI 15	SCI 18
*Lysozyme activity* (*Units/mL*)	653 ± 76^a^	667 ± 57^a^	703 ± 60^ab^	740 ± 63^b^
*Complement activity* (*% inhibition*)	81.4 ± 4.7	81.3 ± 4.6	83.0 ± 5.6	79.3 ± 6.8
*Nitric oxide* (*μM*)	28.9 ± 14.2	34.0 ± 17.5	32.5 ± 9.6	27.3 ± 8.2
*Anti-protease activity* (*% inhibition*)	93.7 ± 2.4^a^	96.2 ± 1.4^b^	97.3 ± 0.9^b^	96.8 ± 0.8^b^
*Ceruloplasmin activity* (*Units/mL*)	63.2 ± 29.0	50.2 ± 23.8	47.8 ± 26.5	58.1 ± 25.4

Data represent mean ± SD, standard deviation. Different letters in the same line denote statistically significant differences (*p* < 0.05).

### 3.5 Histological analysis

The control, SCI 12, and SCI 18 diets caused moderate alterations in the liver (grade 3) (Table 11), while the SCI 15 diet showed mild alterations (grade 2) (Table 11). The histopathological alterations for all the diets included lipid accumulation and pressed nuclei displacement. No signs of steatosis were detected in all the diets. In the anterior gut, the SCI 12, SCI 15, and SCI 18 diets showed minimal histology alterations (grade 1) (Table 11), while the control diet showed mild alterations (grade 2) (Table 11). In the control diet, large lipid droplets in the enterocytes were detected in some cases, while leucocyte infiltration was present in all the diets. Additionally, the control, SCI 12, and SCI 18 diets showed moderate alterations (grade 3) in the posterior gut (Table 11), while the SCI 15 diet resulted in mild alterations (grade 2) (Table 11). Leucocyte infiltration was detected in all the diets, while in the control, SCI 12, and SCI 18 diets, cases of possible enteritis were exhibited. Histological measurements showed that the brush border of the anterior gut was significantly thicker in fish fed the SCI 15 diet, while lipid droplets were bigger in the liver of fish fed the control diet (Table 12).

**TABLE 11:** Severity score (expressed as Grade 0–4) for the observed histopathological alterations of *Dicentrarchus labrax*’s liver, anterior gut and posterior gut.

	Liver	Anterior gut	Posterior gut
Control	3	2	3
SCI 12	3	1	3
SCI 15	2	1	2
SCI 18	3	1	3

**TABLE 12:** Histological measurements in anterior gut and liver of *D. labrax*.

Measurements	Control	SCI 12	SCI 15	SCI 18
Anterior gut brush	2.5 ± 0.6^a^	2.7 ± 0.6^ab^	3.0 ± 0.5^b^	2.8 ± 0.4^ab^
Border width	(38)	(33)	(36)	(30)
Liver lipid droplets	12.8 ± 2.3^a^	10.5 ± 2.3^b^	10.6 ± 2.8^b^	11.4 ± 2.3^b^
Diameter	(70)	(79)	(98)	(106)

Data represent mean ± SD, standard deviation. The number of measurements is represented within brackets. Different letters in the same line denote statistically significant differences (*p* < 0.05).

## 4 Discussion

It is well documented that the high or total replacement of fishmeal in the nutrition of carnivorous fish will adversely affect the growth performance, feed utilization, and immune system of the fish (Torrecillas et al., 2017; Biswas et al., 2020; Basto et al., 2023). Consequently, partial replacement is a possible direction for the sustainability of aquaculture and the welfare of farmed fish. This is the first study conducted for European sea bass where a combination of novel protein ingredients was used for the replacement of fishmeal. The results showed that the novel ingredients consisting of bacterial protein (at inclusion levels of 7.5%, 9.4%, and 11.3% in the diet) and yeast protein (at levels of 3.8%, 4.7%, and 5.6%) with the additional inclusion of algae meal (1% for all experimental diets), did not jeopardize the growth performance indices, whole-body composition, whole-body fatty acid profile, immune response, and intestine morphology. The results even showed that in some parameters there was an improvement compared to the control diet. Specifically, the digestibility of protein was significantly higher for diets with SCI, and similar results were determined for the digestibility of starch.

Bacterial protein has been used in several studies as a replacement for fishmeal. In most of the cases, the bacterial protein was produced by *M. capsulatus* and was tested with different levels of inclusion for several species such as largemouth bass (*Micropterus salmoides*), sea bream (*Sparus aurata*), and spotted seabass (*Lateolabrax maculatus*) (Guo et al., 2022; Zhang et al., 2022; Carvalho et al., 2023; Yu et al., 2023). The inclusion levels of bacterial protein in the aforementioned studies were between 3% and 21.5%. The results detected a nutritional tolerance limit of this protein source for most species which was dependent on fishmeal inclusion. For largemouth bass, the inclusion of bacterial protein in moderate fishmeal diets (26%) reached up to 6% with positive effects on final body weight (Guo et al., 2022) while at the 9% inclusion level, a low feed intake was recorded which was possibly related to palatability issues. In another study for largemouth bass, it was observed that higher fishmeal inclusion (31%) increased the tolerance of the specific species for bacterial protein up to 12.9%, resulting in similar growth parameters as fishmeal diets (Zhang et al., 2022). In sea bream diets, the inclusion of 10% bacterial protein and 5% fishmeal resulted in similar growth parameters as the control fishmeal diet. The same results were observed for whole-body composition, fatty acid, and AA profiles (Carvalho et al., 2023). In a research study conducted on spotted sea bass, the evidence showed that the inclusion of bacterial protein can reach 14% with the addition of 21% fishmeal without depressing growth parameters (Yu et al., 2023).

Additionally, several studies have evaluated the inclusion of bacterial protein in species with high dietary protein requirements like rainbow trout (*Oncorhynchus mykiss*), Japanese yellowtail (*Seriola quinqueradiata*), turbot (*Scophthalmus maximus* L.), and red sea bream (*Pagrus major*) (Biswas et al., 2020; Biswas et al., 2021; Rajesh et al., 2022; Zheng et al., 2023). A study conducted by Rajesh et al. (2022) for rainbow trout, concluded that the bacterial protein can be included safely at 12.5% when fishmeal inclusion is 37%. Growth parameters were negatively affected as bacterial protein increased above 12.5% and the same results were observed for HSI. The digestibility of fat decreased as bacterial protein increased and the same trend was detected for protein and fat content of the whole body. Biswas et al. (2021) tested the replacement of fishmeal with bacterial protein up to 100% for yellowtail kingfish and the results showed that for this specific species, fishmeal replacement is possible up to 25% (17% bacterial protein, 51% fishmeal). The results showed a negative trend for growth parameters and protein digestibility when levels of bacterial protein increased above 25%. During a second trial, the authors replaced 20%, 25%, and 30% of the fishmeal with bacterial protein with the addition of an attractant to identify possible palatability issues. The results showed no differences among treatments for growth parameters, whole body composition, and digestibility of nutrients, proving that replacement of fishmeal can reach 30% if an attractant is incorporated. Zheng et al. (2023) evaluated the inclusion of bacterial protein in diets for turbot at inclusion levels from 9.2% to 61.1% in the diet. The study revealed positive effects on growth performance indices up to 18.3% inclusion. Above this level, a negative trend was observed for growth parameters, digestibility of nutrients, and essential fatty acids in muscle. For red sea bream, a trial by Biswas et al. (2021) was conducted where bacterial protein was included in the experimental diets from 12% up to 36% and fishmeal levels were from 56% to 28%. The outcome showed that the inclusion of up to 12% is feasible with 56% fishmeal in the diet. The rest of the inclusion levels caused adverse effects on growth parameters. Furthermore, a significant reduction in whole body lipid content and in EPA fatty acid for diets with higher inclusion levels of bacterial protein was detected.

Besides bacterial protein, several studies evaluated the replacement of fishmeal with yeast protein ingredients. For rainbow trout, a study was performed by Vidakovic et al. (2020) aiming for the replacement of fishmeal with yeast *S. cerevisiae* at levels of 10%–32%. The results showed that the inclusion of yeast up to 21% is possible with no detrimental effects on growth performance, nutrient digestibility, and intestinal health. In channel catfish (Hao et al., 2022), the inclusion of yeast at 2% improved growth performance and feed utilization. Additionally, improved immune response and sustained intestinal health were determined. Intact and extracted yeast have been evaluated for Arctic charr (*Salvelinus alpinus*), replacing 40% fishmeal on a crude protein basis. The results were positive concerning the intact yeast but the extracted yeast negatively affected growth performance. The digestibility of nutrients, however, was similar to the reference diet (Vidakovic et al., 2016). For European sea bass, a study conducted by Dawood et al. (2021) indicated that the incorporation of *S. cerevisiae* can enhance growth performance, immune genes, and the antioxidative status of the experimental population when supplementation is 0.1%–0.2%. A product with inactivated dry yeast (*S. cerevisiae*) was evaluated for Nile tilapia by Yossa et al. (2021) by replacing fishmeal from 2% to 100%. The overall results showed that for this specific species, fishmeal can be totally replaced by this yeast product with improvement in feed and nutrient utilization. Besides *S. cerevisiae,* increasing attention has been given to other yeast, such as *Candida utilis* and *Kluyveromyces marxianus,* as protein sources to replace fishmeal in studies concerning Atlantic salmon. In 2013; Øverland et al. (2013) replaced 40% of crude protein from fishmeal with the aforementioned yeasts and *S. cerevisiae*. The results revealed that *C. utilis* and *K. marxianus* can be used as alternative protein sources for Atlantic salmon with no negative effects. For Atlantic salmon; Øvrum Hansen et al. (2019), evaluated the replacement of fish and soybean meal with *C. utilis* with promising results for growth performance. Overall, the results showed different tolerances of yeast protein inclusion in the diets of different species. However, the fact that the nutritional value of yeast depends heavily on culture conditions, yeast species, and processing must be considered (Agboola et al., 2022).

Numerous studies have been published on the effect of microalgae single-cell proteins (SCPs) on the growth performance, digestibility, health, and immune response of various species. In European sea bass, partial replacement of fishmeal by *Nannochloropsis oceanica* was evaluated (Batista et al., 2020), resulting in similar final body weight, FCR, and digestibility of protein compared to the reference diet. Different species of microalgae dried biomass (*Tisochrysis lutea* and *Tetraselmis suecica*) have been tested for the same species in low fishmeal and fish oil diets (Cardinaletti et al., 2018), showing that 45% of crude protein and 36% of lipids can be replaced with no negative effects on growth performance and feed utilization. In red drum (*Sciaenops ocellatus*) feeds, a microalgae dietary combination of *Arthrospira sp*. and *Schizochytrium limacinum* resulted in similar growth parameters, feed utilization and body condition indices compared to the reference diet. Additionally, the data showed that *S. limacinum* meal can be used as main source of lipid for the red drum (Perez-Velazquez et al., 2018). A trial combining three microalgae species (*Nannochloropsis* sp., *Isochrysis* sp., and *Schizochytrium* sp.) was performed for rainbow trout (Sarker et al., 2020), concluding that *Isochrysis* sp., and *Schizochytrium* sp. are effective candidates for DHA supplementation in trout diets. *Schizochytrium* sp. oil was also evaluated as a fish oil replacement for Atlantic salmon and was suggested as a successful total replacement of conventional fish oil (Tibbetts et al., 2020). 

A few studies have been performed to evaluate combined sources of SCI (Rajesh et al., 2022; Lu et al., 2023; Yang et al., 2023). In this study, a different approach to diet formulation was employed which led to promising results. The inclusion levels were 7.5%, 9.4%, and 11.3% of bacterial protein, 3.8%, 4.7%, and 5.6% of yeast meal and 1% algae meal in all the SCI including diets, respectively. The growth performance revealed higher final body weight, weight gain, specific growth rate (SGR), and lower FCR for all inclusion levels of SCI even though consumption was the same for all experimental groups. The inclusion of krill meal in the diets minimized possible palatability issues due to the tested ingredients as discussed in a number of studies (Biswas et al., 2020; Biswas et al., 2021; Rajesh et al., 2022). The digestibility of protein was significantly higher for all diets compared to the fishmeal diet. This result cannot be correlated with lower digestibility of fish meal trimmings compared to premium fishmeal as other studies reported (Dam et al., 2019) since all the diets included this type of fishmeal. However, this result is in accordance with the findings of Guo et al. (2022) in largemouth bass where all dietary inclusion levels (3%, 6%, and 9%) of bacterial protein showed higher digestibility of protein compared to the fishmeal diet. The same trend was detected for the digestibility of starch where all bacterial protein diets demonstrated significantly higher digestibility coefficients of starch compared to the fishmeal diet. In the current study, whole-body EPA and DHA levels showed a linear decrease as SCI increased in the dietary treatment. The same linear trend was observed also by other authors for red sea bream and turbot (Biswas et al., 2021; Zheng et al., 2023). The fatty acid profile of the diets reflected the whole-body fatty acid profile of the experimental groups (r = 0.99) as was well established by Sargent et al. (1995) and Fountoulaki et al. (2009).

All the diets in the current study met the EAA requirements of European sea bass. Lysine levels in the experimental treatments were in excess compared to the control diet while methionine was lower for all the SCI diets. Additionally, arginine levels in feeds with SCI were lower by 11%–15% from the control diet, however, this resulted in similar AA carcass content. Arginine can increase the activity of several intestinal enzymes and promote nutrient absorption (Li et al., 2022) and this could be associated with the higher digestibility of protein observed in treatments with SCI. Even though arginine levels in the experimental feeds were lower compared to the control, arginine carcass content was similar for the experimental population and this may enhance the claim that bacterial protein provides a higher nutritional value of this amino acid The low-level inclusion of SCI resulted in equal EAA content to the control group, while at higher inclusion levels, EAA content was lower Although the content of most AA in the SCI 12 and SCI 1 feeds 5 were deficient compared to the control diet, the carcass content resulted in similar levels, indicating higher availability of AAs for these diets. The yeast protein tested is a proprietary extract of *S. cerevisiae* which contains approximately 5% nucleotides by dry weight (Fegan, 2006), and the benefits of which are well documented (Craig and McLean, 2005; Fegan, 2006). On the contrary, Riche et al. (2017) found poor protein digestibility and low AA availability in Florida pompano *Trachinotus carolinus* when fed a diet with 7.5% yeast protein. Nucleic acids in yeast comprise 13%–23% of total N (Oliva-Teles et al., 2006). Nucleotides not taken up by the mucosa appear as undigested fecal N and decrease protein digestibility estimations, although, in the current study, this was not the case as protein digestibility was rather high at all SCI inclusion levels. Gut microbiota may also be affected by the inclusion of yeast at the two moderate inclusion levels, as reported in human nutritional studies (Portune et al., 2016). Beneficial bacteria can synthesize EAA in the gut of the fish, explaining the similar amounts of EAA found in the carcass of the fish for the low and moderate inclusion levels of SCI.

SCPs are indeed rich in proteins but they also contain DNA, RNA, mannan-oligosaccharides (MOS), and polysaccharides such as β-glucans, chitin, and nucleotides that may act as prebiotics and/or have immunostimulating activities in many fish species (Hoseinifar et al., 2015; Glencross et al., 2020). A 5%–15% dietary inclusion of methanotroph bacterial meal (*M. capsulatus*) did not significantly affect the immune parameters of white shrimps (*Penaeus vannamei*) but they better resisted a bath challenge with *Vibrio parahaemolyticus* (Jintasataporn et al., 2021). In fish, 1.7%–8.5% of dietary single cells bacteria significantly increased Ig titer but decreased cytokines expression in tilapia (*Oreochromis niloticus*) (Chama et al., 2021). In American eel (*Anguilla rostrate*), 12%–18% methanotroph bacteria significantly increased the complement C3 expression but the alternative pathways of the complement and lysozyme activities were not affected by dietary SCI. However, intestine villi height and density and muscular thickness significantly decreased at these levels while 6% did not affect these parameters (Lu et al., 2023). Yeasts, as potential sustainable ingredients, have shown positive effects on the immune system of fish. In Nile tilapia, a hydrolyzed yeast protein (*Rhodotorula mucilaginosa*) at 0.5%–1% dietary inclusion has been shown to stimulate both lysozyme activity and complement C3 content (Chen et al., 2019). Concerning autolyzed brewer yeast extract, a dietary dose of 1%-2% did not significantly affect the lysozyme or the respiratory burst activity of head-kidney leucocytes of hybrid striped bass (*Morone chrysops* × *M. saxatilis*), however, an improvement in resistance to mycobacterial infection was observed (Li and Gatlin, 2005). Gilthead seabream was immunostimulated by 0.1%–1% dietary whole yeast (increased phagocytosis, respiratory burst, and cytotoxic activity) while complement activity was not affected (Ortuño et al., 2002). In Labeo rohita, *the* dietary inclusion of 1% yeast extract enhanced the total leucocyte count and blood respiratory burst activity (Andrews et al., 2011). Dietary doses of yeast ranging from 1%-8% increased the leukocyte counts (lymphocytes, monocytes, and neutrophils) in pirarucu, *Arapaina gigas* (Hoshino et al., 2020) but did not significantly stimulate the phagocytosis or lysozyme activity of Nile tilapia (Berto et al., 2016). On the contrary, a dietary dose of 4% yeast significantly increased the lysozyme activity of pikeperch (*Sander lucioperca*) together with cellular immunity parameters such as respiratory burst activity, phagocytposis, and proliferation) while ceruloplasmin activity was unchanged (Kowalska et al., 2015). Finally, a dietary dose of 4%-8% yeast protein had a stimulating effect on the blood respiratory burst activity and on the liver morphology, reducing steatosis and fat degeneration observed in European labrax (*D. labrax*) while no effect was observed on the gut histology (Panagiotidou et al., 2016). In the current study, the immunostimulating activity of SCPs was observed through a significantly increased lysozyme activity at the two highest inclusion levels of SCI, as has been previously obtained with dietary yeast in pikeperch and tilapia (Kowalska et al., 2015; Chen et al., 2019). This suggests a better defensive potential of those fish fed SCI against Gram-positive bacterial pathogens. Trypsin inhibition activity was enhanced at all 3 dietary levels tested which suggests that fish fed SCI may better counteract the evasion process of potential pathogens. Additionally, the antibacterial activity of the complement against a Gram-negative bacterium was not affected by the dietary inclusion of SCI in contrast with gene expression results obtained in American eel and tilapia (Lu et al., 2023; Chen et al., 2019). However, this result was in agreement with complement activity measured in striped bass and gilthead seabream (Ortuno et al., 2002; Li and Gatlin, 2005). The ceruloplasmin remained unchanged as already shown in pikeperch (Kowalska et al., 2015).

Mannan-oligosaccharides (MOS) and polysaccharides such as β-glucans, chitin, and nucleotides contained in SCIs may directly modulate the immune system of fish and/or may act indirectly as prebiotics modulating the microbiota which, in turn, interact with the immune system of the fish. Furthermore, SCI were shown to also affect the microbiota of rainbow trout (Nyman, 2016) which could explain some of the immunomodulation observed in fish fed these ingredients.

A liver and intestine histological examination is important when evaluating the effectiveness of a new fish diet. Functionally, the anterior region of the gut is the primary site for nutrient uptake. The brush border (microvilli) of the apical surface of the enterocytes increases the area of absorption (Berillis et al., 2017). The liver has the ability to oxidize fatty acids and an excess of lipids in the diet can be detected by the presence of lipid droplets in the hepatocytes (Berillis et al., 2017). There is a lack in the literature regarding the effects of bacterial SCP on liver and gut histology in fish species. Marchi et al. (2023), working with sea bream, explored the efficacy of dietary inclusion levels of bacterial SCP to replace plant protein sources. Gut histology revealed no histopathological changes due to SCP in sea bream intestines, indicating that bacterial SCP up to 20% could replace soy-derived proteins, without altering the anatomic structure of the intestine. Dietary SCI have shown the ability to prevent soybean meal-induced enteritis in Atlantic salmon probably due to the cell wall fraction of the bacterium *M. capsulatus* (Romarheim et al., 2011; Romarheim et al., 2013). In American ell (*A. rostrate*), 12%–18% methanotroph bacteria in the diet significantly decreased intestine villi height, density, and muscular thickness while 6% did not affect these parameters significantly (Lu et al., 2023). Yang et al. (2023) showed that by replacing 50%–70% of dietary fishmeal with *Clostridium autoethanogenum* in the diets of largemouth bass (*M. salmoides*), the villi height and muscle thickness decreased significantly. On the contrary, Ma et al. (2022) showed that *C. autoethanogenum* protein replacing up to 50% of fishmeal in largemouth bass (*M. salmoides*) diets did not affect the hepatic structure, hepatocytes, the villi nor the columnar epithelial cells of the distal intestine. Concerning yeast protein extracts, a dietary dose of 4%-8% had a protective effect on the liver morphology, reducing steatosis and fat degeneration in European sea bass (*D. labrax*) while no effect was observed on the gut histology (Panagiotidou et al., 2016). In the present study, lipid accumulation and pressed nuclei displacement of the hepatocytes were detected for all diets (control, SCI 12, SCI 15, and SCI 18) in the liver, possible due to typical liver histomorphology in cultured fish. Fish fed the control diet had bigger lipid droplets in their hepatocytes than the fish fed with the SCI diets. As no steatosis was detected, bigger lipid droplets in the control diet do not correspond to lower lipid digestibility or higher fat content in the fish body. The most severe histopathological alterations were in the posterior gut. Inflammation signs (leucocyte infiltration) were detected in all diets, while in the control, SCI 12, and SCI 18 diets there were cases of possible enteritis. Enteritis can be caused when carnivorous fish are fed diets with high levels of plant-based proteins (Zhang et al., 2013) but this was not the case since SCI totally replaced soya bean meal. Leucocyte infiltration was also detected in the anterior gut. The brush border of the anterior gut was thicker in fish fed the SCI 15 diet, probably indicating higher nutrient absorption. This fact can reflect the tendency of higher SGR and weight gain which were detected in fish fed the SCI 15 diet. The severity scores of the histological observations showed a tendency for an improved anterior and posterior gut and liver morphology when fish were fed the SCI diets compared to control diet fed fish, explaining some of the immunomodulation observed in the fish fed the SCI diets.

Despite the fact that SCI are produced in a sustainable way that addresses environmental and social challenges, demand and production are still low, which is reflected in their price. Many research studies have recently demonstrated the positive effects of these ingredients in livestock production, a fact that will drive up their production and availability to levels where their economic profitability can be more accurately assessed.

In conclusion, the overall performance of European sea bass juveniles showed that the incorporation of single-cell ingredients could be beneficial in improving most of the parameters evaluated. Specifically, fishmeal trimmings can be reduced by up to 7%, plant ingredients up to 24%, and fish oil from trimmings by 6% without compromising key performance indicators. This approach to feed formulation can be sustainable for organic aquaculture, promoting higher nutrient digestibility and maintaining protein content in the whole body of experimental fish while depositing similar levels of amino acids as a commercial-type organic diet. The inclusion of 15% SCI in the diet (bacterial: yeast: algae—9.4: 4.7: 1) proved more efficient in most parameters evaluated for European sea bass. Further research to determine the optimum inclusion level of SCI for adult European sea bass is necessary to evaluate their suitability to all stages of the production cycle of the species.

## Data Availability

The original contributions presented in the study are included in the article/Supplementary Material, further inquiries can be directed to the corresponding author.
