# Supplementary material for: Fishmeal, plant protein, and fish oil substitution with single-cell ingredients in organic feeds for European sea bass (Dicentrarchus labrax)
Source: Front Physiol. 2023 May 15;14:1199497. doi: 10.3389/fphys.2023.1199497 (PMC10225740; doi:10.3389/fphys.2023.1199497)
Supplement: Supplementary file 1 [file DataSheet1.docx]

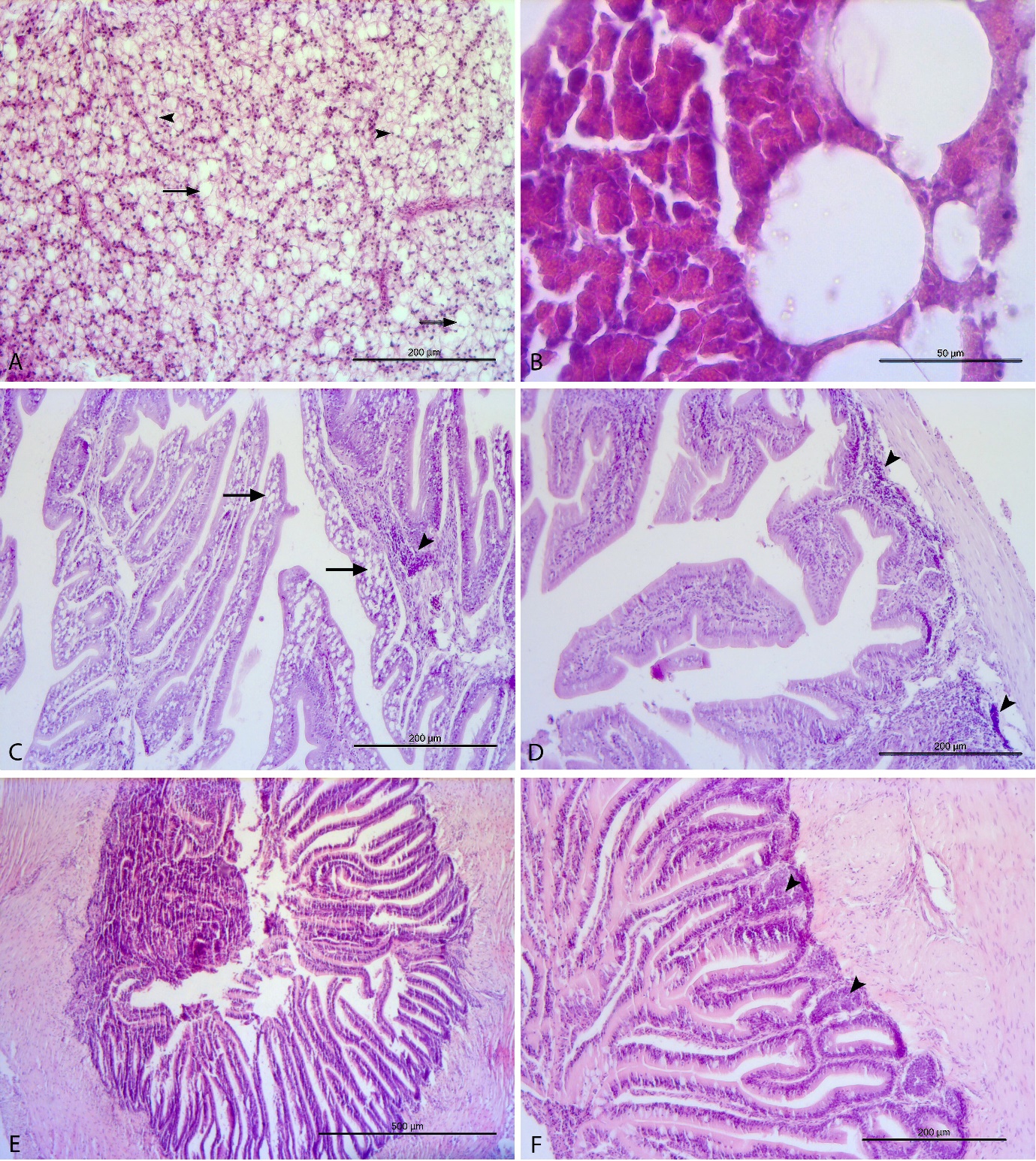


**Figure 1**. Liver, anterior gut, and posterior gut histopathology. (A) Liver. SCI 18 diet group. Big lipid droplets in the hepatocytes (arrows). Some of the nuclei (arrowheads) are pushed to the edge of the cells by lipid droplets. (B) Liver. SCI 12 diet group. Big lipid droplets at pancreatic islet. (C) Anterior gut. Control diet group. Big lipid droplets in the enterocytes of the anterior gut (arrows) and leucocyte infiltration (arrowhead). (D) Anterior gut. SCI 15 diet group. Leucocyte infiltration (arrowheads). (E) Posterior gut. SCI 12 group. Intestinal folds fusion. Possible enteritis. (F). SCI 15 group. Leucocyte infiltration (arrowheads).
